# Supplementary material for: Sulfate residuals on Ru catalysts switch CO2 reduction from methanation to reverse water-gas shift reaction
Source: Nat Commun. 2024 Nov 2;15:9478. doi: 10.1038/s41467-024-53909-8 (PMC11531589; doi:10.1038/s41467-024-53909-8)
Supplement: Supplementary file 1 — Supplementary Information [file 41467_2024_53909_MOESM1_ESM.pdf]

Supporting Information

Sulfate residuals on Ru catalysts switch CO_2 reduction from methanation to reverse water-gas shift reaction

Min Chen[1], Longgang Liu[2], Xueyan Chen[1], Xiaoxiao Qin[1], Jianghao Zhang[1], Shaohua Xie[3], Fudong Liu[3*], Hong He[1,4], Changbin Zhang[1,4*]

[1] State Key Joint Laboratory of Environment Simulation and Pollution Control, Research Center for Eco-Environmental Sciences, Chinese Academy of Sciences, Beijing 100085, China

[2] School of Chemistry and Chemical Engineering, Qufu Normal University, Qufu 273165, China

[3] Department of Chemical and Environmental Engineering, Bourns College of Engineering, Center for Environmental Research and Technology (CE-CERT), Materials Science and Engineering (MSE) Program, University of California, Riverside, California 92521, United States

[4] University of Chinese Academy of Sciences, Beijing 100049, China

*Corresponding authors: fudong.liu@ucr.edu (F. Liu); cbzhang@rcees.ac.cn (C. Zhang)

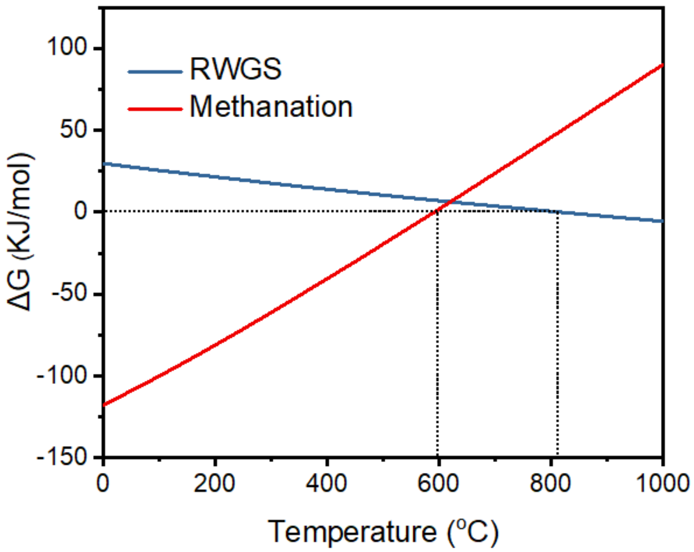

Supplementary Fig. 1 | The changes in Gibbs free energy of RWGS and methanation reactions at different temperatures.

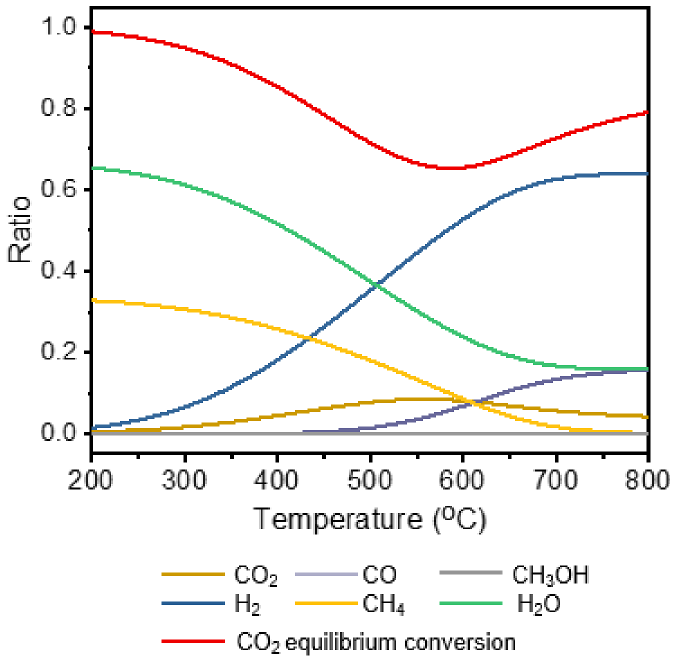

Supplementary Fig. 2 | Influence of temperature on the thermodynamic equilibrium of the CO_2 hydrogenation reaction at 1 bar.

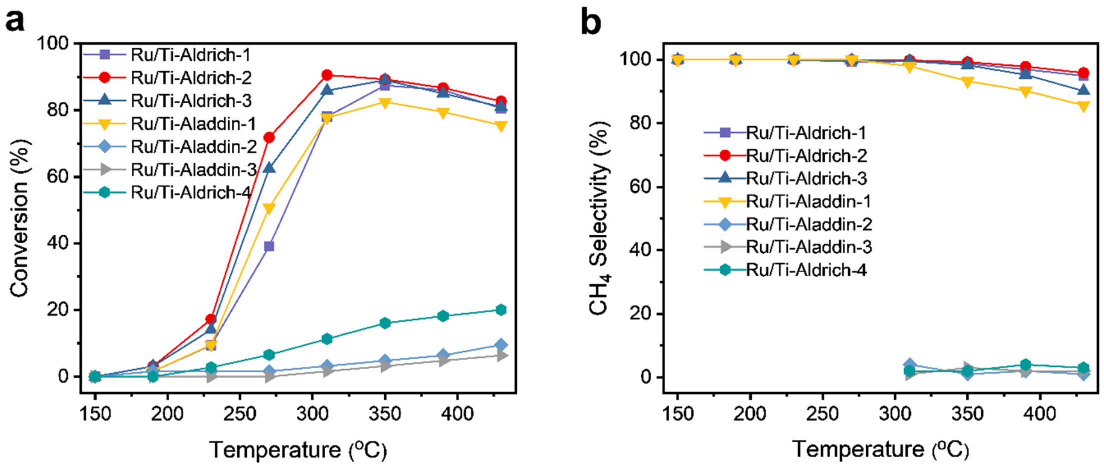

Supplementary Fig. 3 | (a) Temperature-dependent CO_2 conversions and **(b)** CH_4 selectivity on Ru/TiO_2 catalysts prepared using different commercial anatase TiO_2 supports.

Supplementary Table 1 | ICP-MS results of the S content in different Ru/TiO_2 catalysts.

Sample	S mass content (wt.%)
Ru/Ti-Aldrich-1	0.01%
Ru/Ti-Aldrich-2	0.01%
Ru/Ti-Aldrich-3	0.03%
Ru/Ti-Aldrich-4	0.58%
Ru/Ti-Aladdin-1	0.02%
Ru/Ti-Aladdin-2	0.69%
Ru/Ti-Aladdin-3	0.10%
Ru/Ti-S-R	0.21%
Ru/Ti-S-AR	0.22%
Ru/Ti-S-R-AR	0.20%
Ru/Ti-R	0.01%
Ru/Ti-AR	0.02%
Ru/Ti-R-AR	0.01%
Ru/R-S-R	0.21%
Ru/R-S-AR	0.28%
Ru/R-R	0.02%
Ru/R-AR	0.01%

Supplementary Table 2 | Ru contents, Ru particle size and Ru dispersion on different Ru/TiO_2 catalysts.

	Ru contents [a]	Ru particle diameter [b]	Ru crystallite size [c]	Ru dispersion [d]
Ru/Ti-R	4.95%	2.9 nm	10.5 nm	25.2%
Ru/Ti-AR	4.98%	5.5 nm	13.1 nm	12.1%
Ru/Ti-S-R	4.94%	2.6 nm	11.3 nm	26.7%
Ru/Ti-S-AR	4.95%	5.7 nm	15.4 nm	10.6%

[a] Determined from ICP results.

[b] Calculated from HAADF-STEM images.

[c] Calculated from XRD.

[d] Calculated from CO chemisorption results.

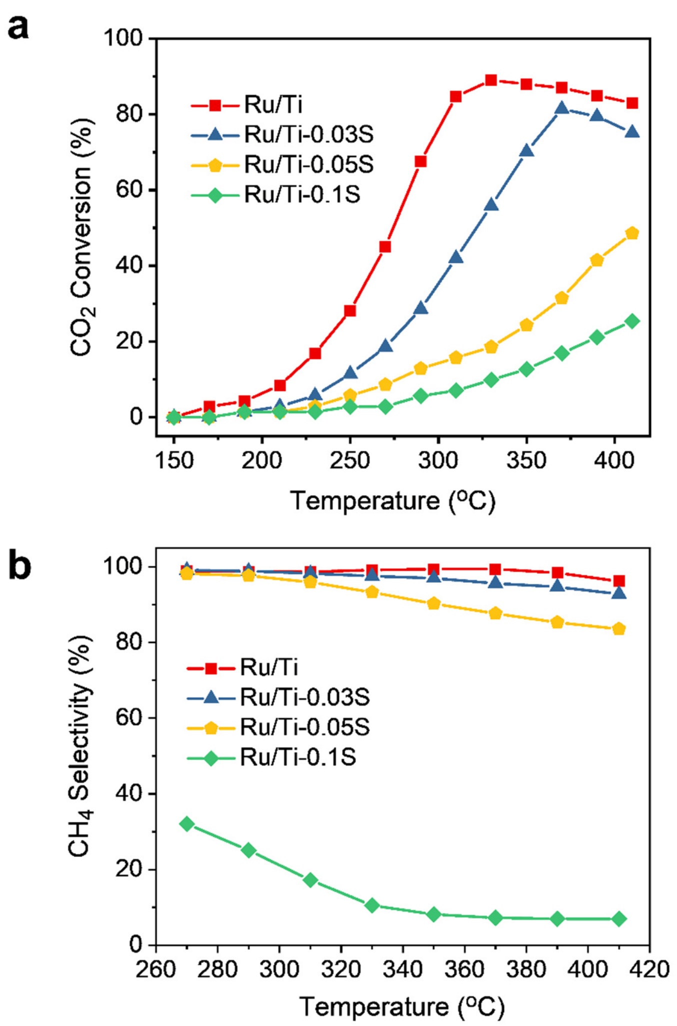

Supplementary Fig. 4 | (a) Temperature-dependent CO_2 conversions and **(b)** CH_4 selectivity on Ru/TiO_2 catalysts with different mole ratios of S/Ru.

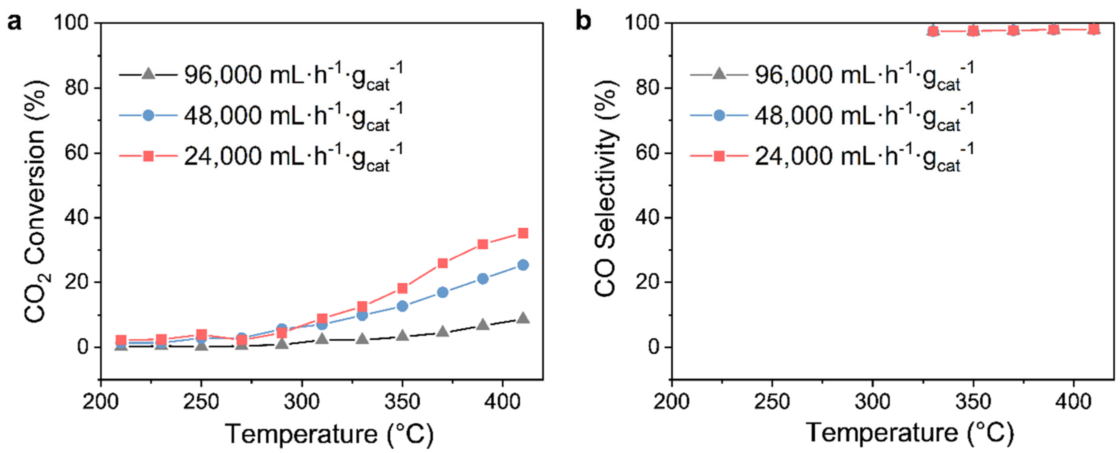

Supplementary Fig. 5 | Temperature-dependent CO_2 conversions and CO selectivity on Ru/Ti-S-AR catalysts when changing the weight hourly space velocity.

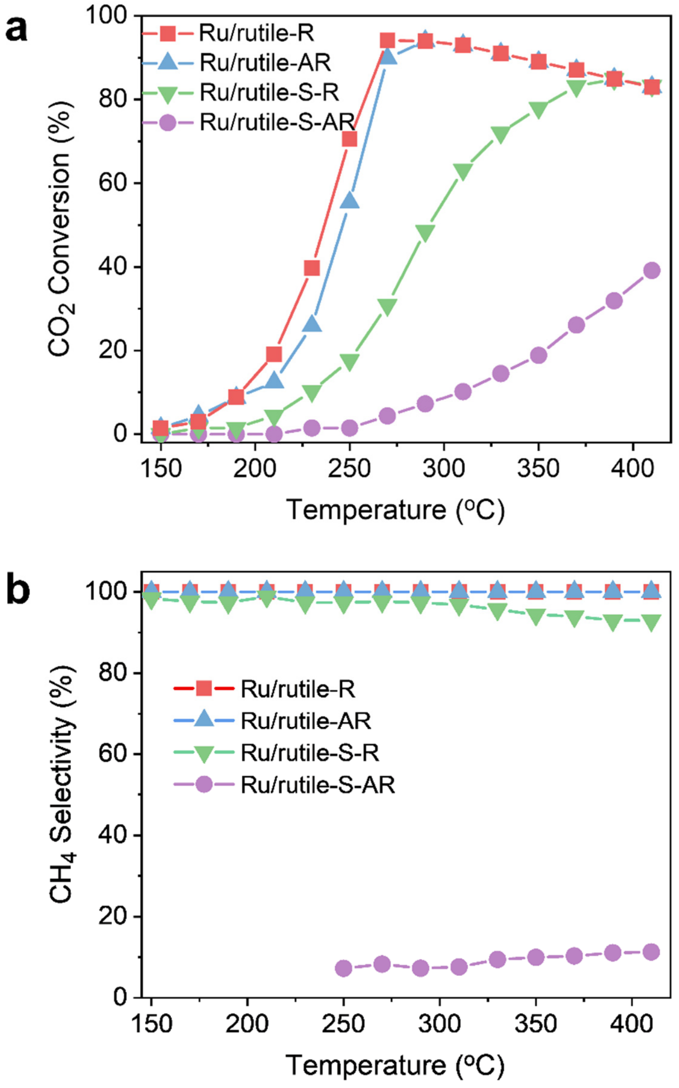

Supplementary Fig. 6 | (a) Temperature-dependent CO_2 conversions and **(b)** CH_4 selectivity on Ru/rutile catalysts containing or not containing SO_4^{2-}.

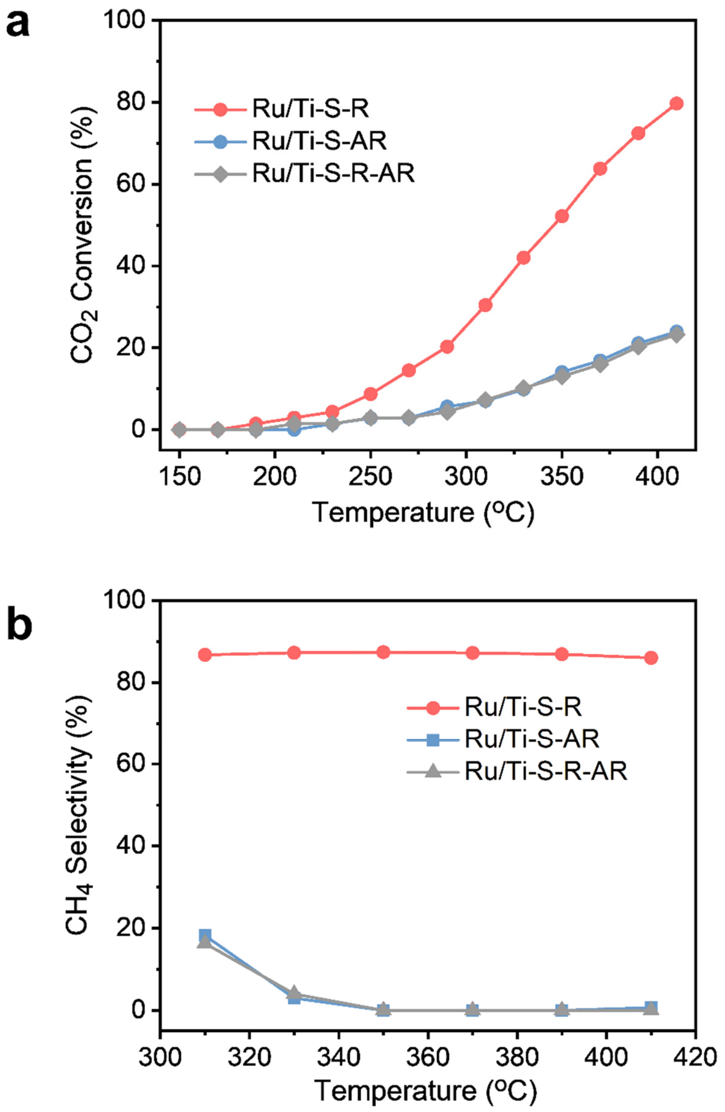

Supplementary Fig. 7 | (a) Temperature-dependent CO_2 conversions and **(b)** CH_4 selectivity on Ru/Ti-S catalyst with air and/or H_2 pretreatment.

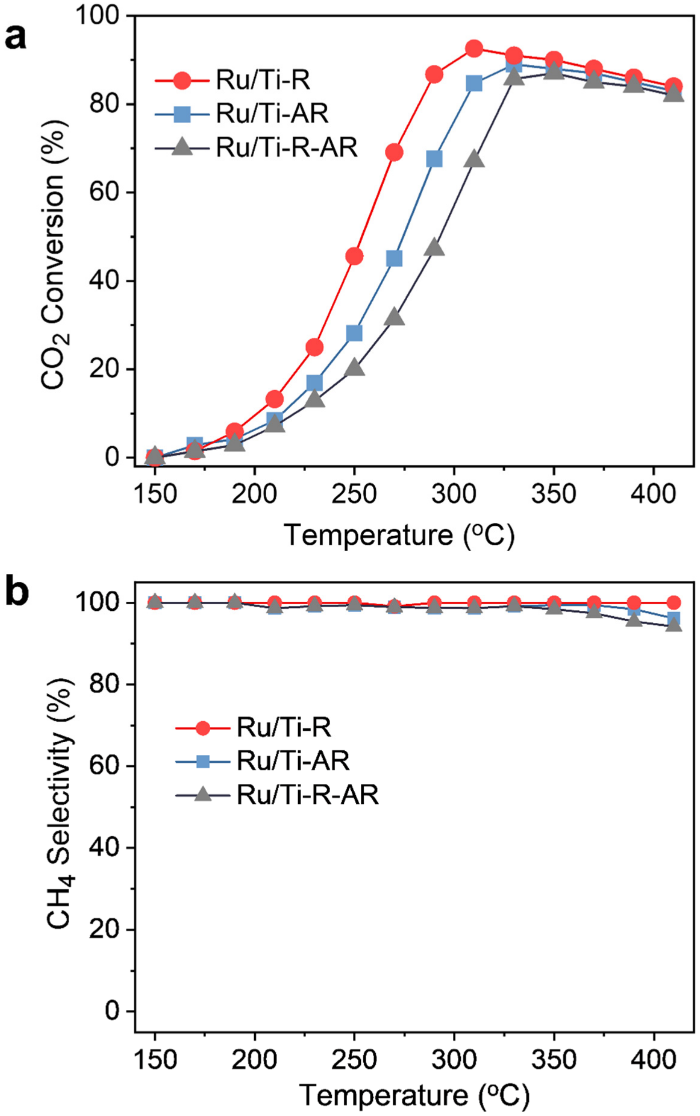

Supplementary Fig. 8 | (a) Temperature-dependent CO_2 conversions and (b) CH_4 selectivity on Ru/Ti catalyst with air and/or H_2 pretreatment.

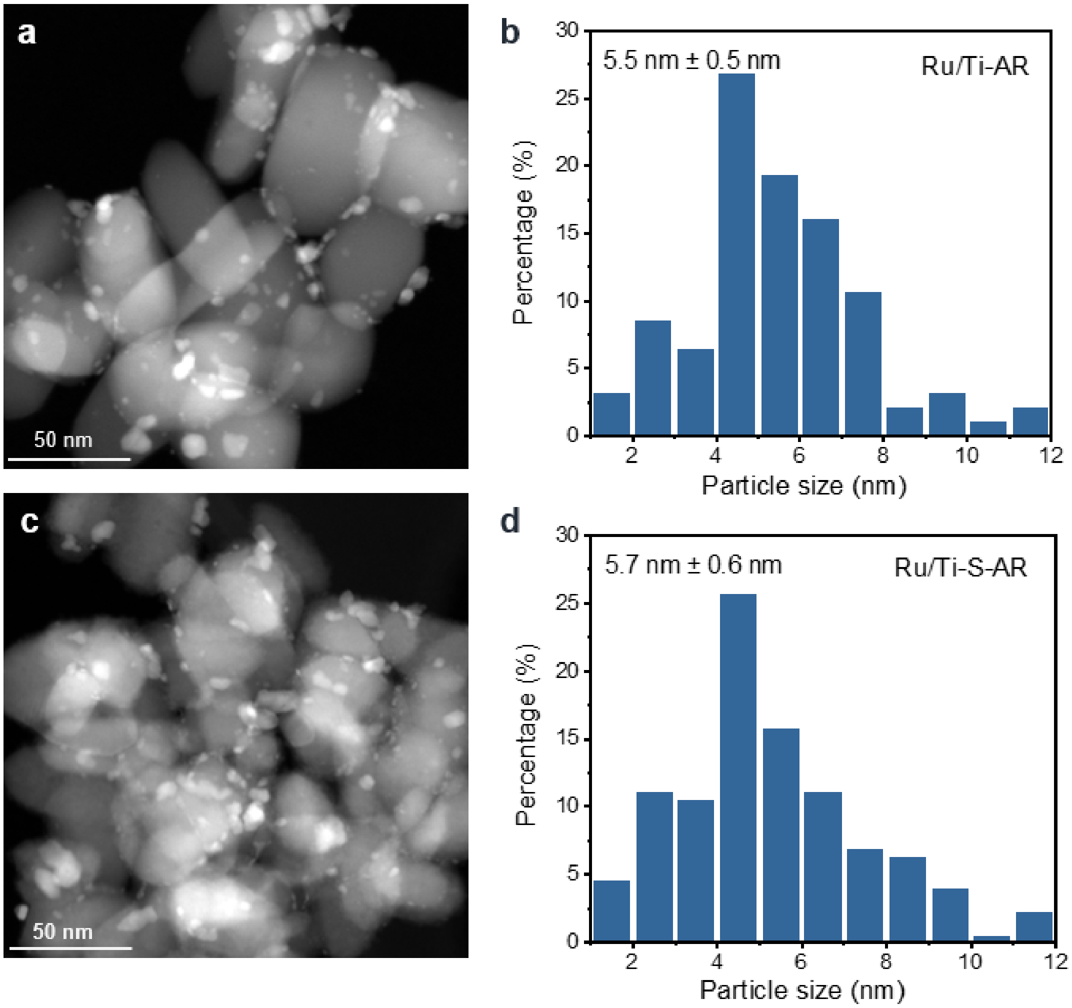

Supplementary Fig. 9 | (a) HAADF-STEM image and **(b)** Ru size distribution of Ru/Ti-AR; **(c)** HAADF-STEM image and **(d)** Ru size distribution of Ru/Ti-S-AR.

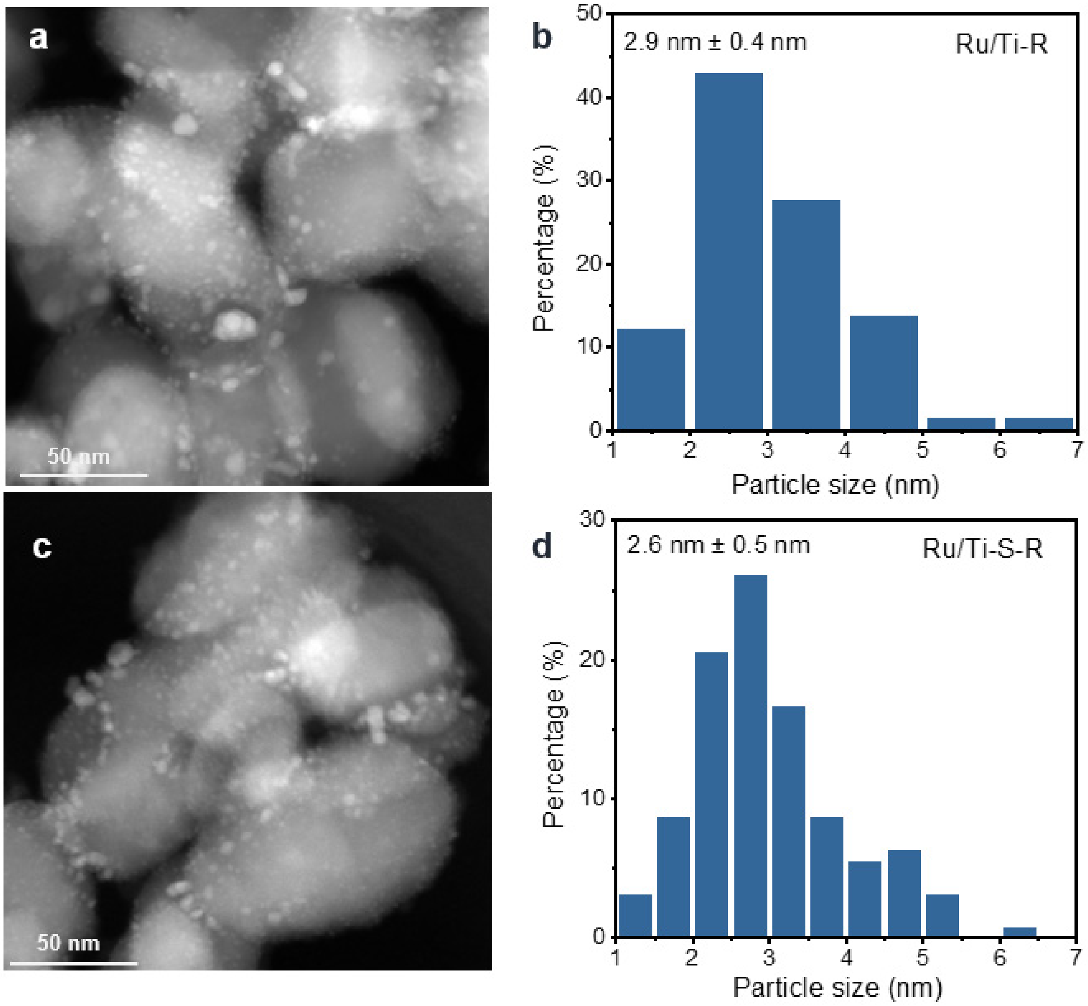

Supplementary Fig. 10 | (a) HAADF-STEM image and **(b)** Ru size distribution of Ru/Ti-R; **(c)** HAADF-STEM image and **(d)** Ru size distribution of Ru/Ti-S-R.

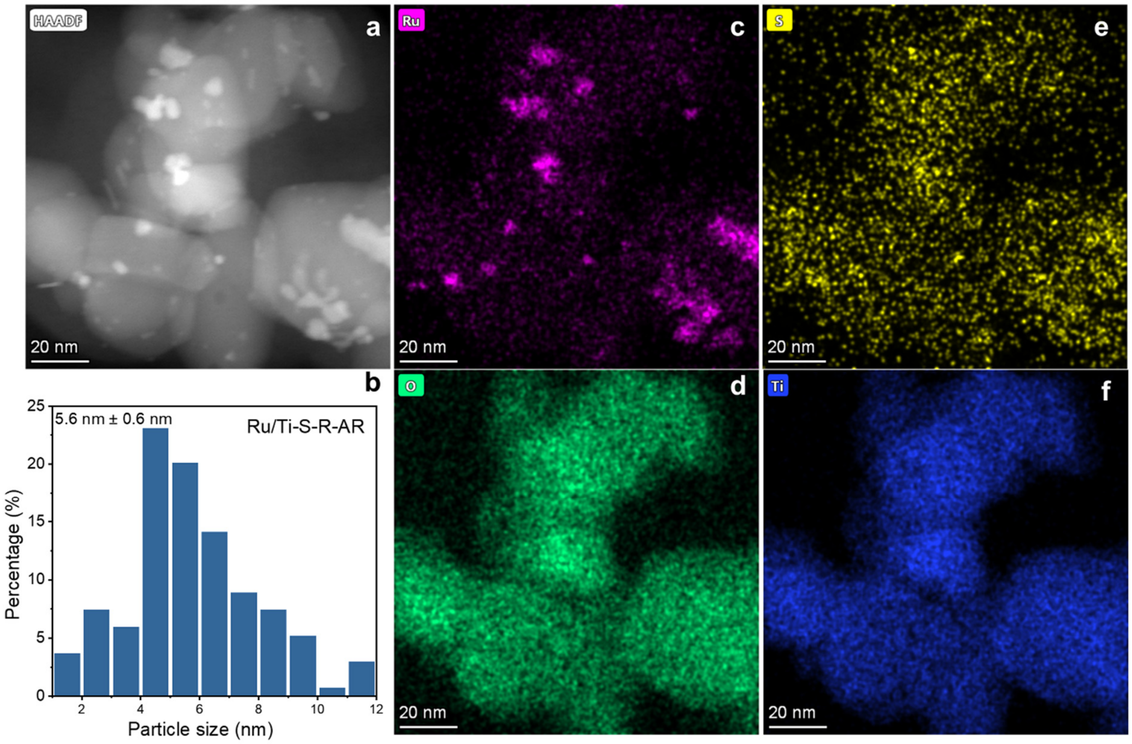

Supplementary Fig. 11 | (a) HAADF-STEM image, **(b)** Ru size distribution, and **(c)-(f)** EDX mapping images of Ru, S, O, and Ti on Ru/Ti-S-R-AR.

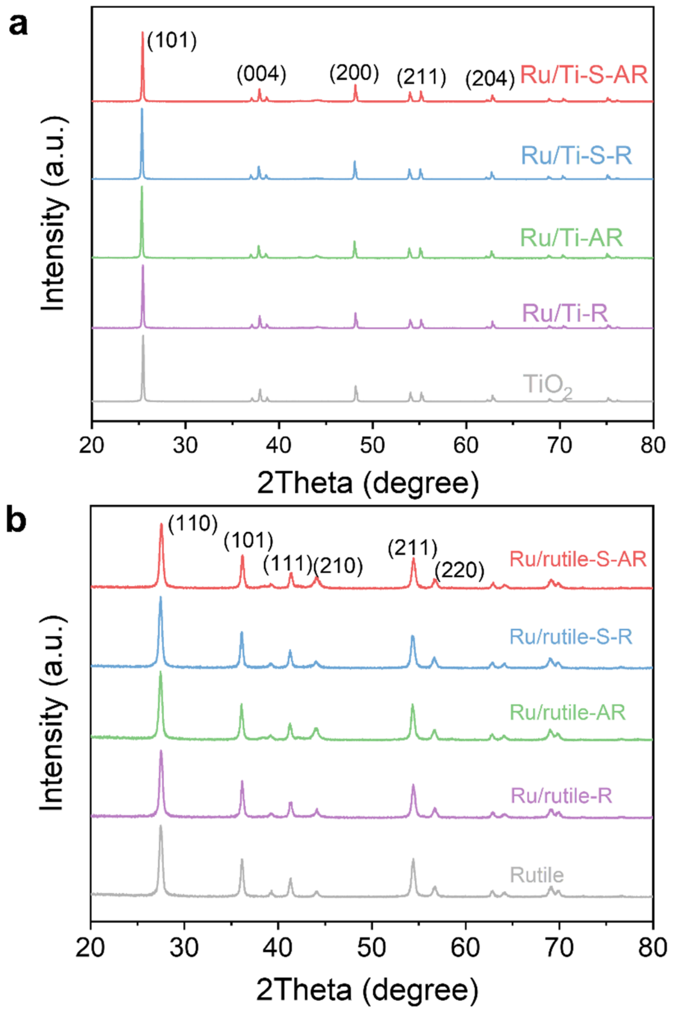

Supplementary Fig. 12 | Powder X-ray diffraction patterns of **(a)** TiO_2, Ru/Ti-R, Ru/Ti-AR, Ru/Ti-S-R and Ru/Ti-S-AR; **(b)** Rutile, Ru/rutile-R, Ru/rutile-AR, Ru/rutile-S-R, Ru/rutile-S-AR.

Note: The powder X-ray diffraction patterns of the prepared Ru/Ti and Ru/rutile samples are shown in Supplementary Fig. 12. The most prominent diffraction peaks were detected at 25.3 and 48.1 °, which was related to the (101) and (200) planes of anatase. For all Ru/rutile samples, 27.5 and 36.2 ° were the most prominent diffraction peaks, which were ascribed to the (110) and (101) planes of rutile. Hence, sulfate species did not induce the restructuration of the support. In addition, the diffraction peak at $2\theta = 44.0$ ° on Ru/Ti-R, Ru/Ti-AR, Ru/Ti-S-R and Ru/Ti-S-AR could be due to the diffraction of the (101) planes of Ru. We calculated the average Ru crystallite size using the Scherrer equation and the results were 10.5, 13.1, 11.3, and 15.4 nm, respectively. It should be pointed out that the Ru peaks were overlapped by the (210) plane of rutile TiO_2. Hence, we did not calculate the average crystallite size of Ru species on Ru/rutile catalysts by XRD.

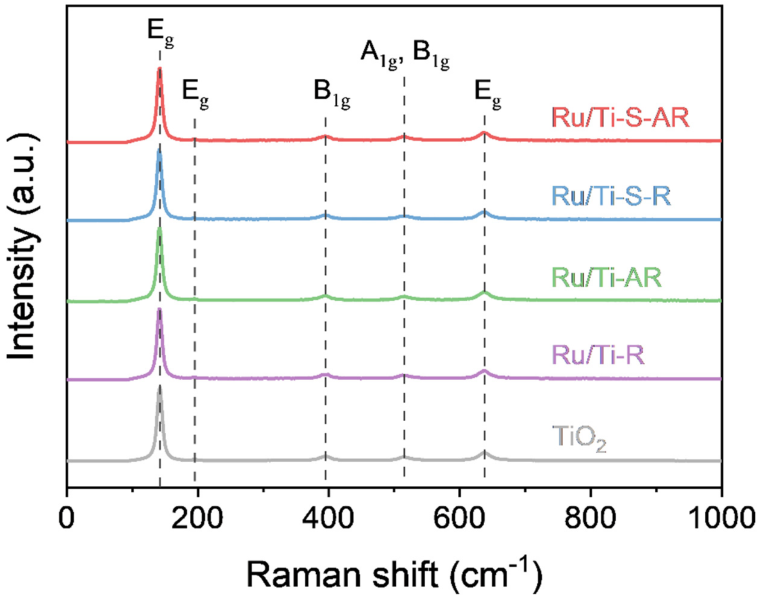

Supplementary Fig. 13 | The Raman spectra of TiO_2, Ru/Ti-R, Ru/Ti-AR, Ru/Ti-S-R and Ru/Ti-S-AR.

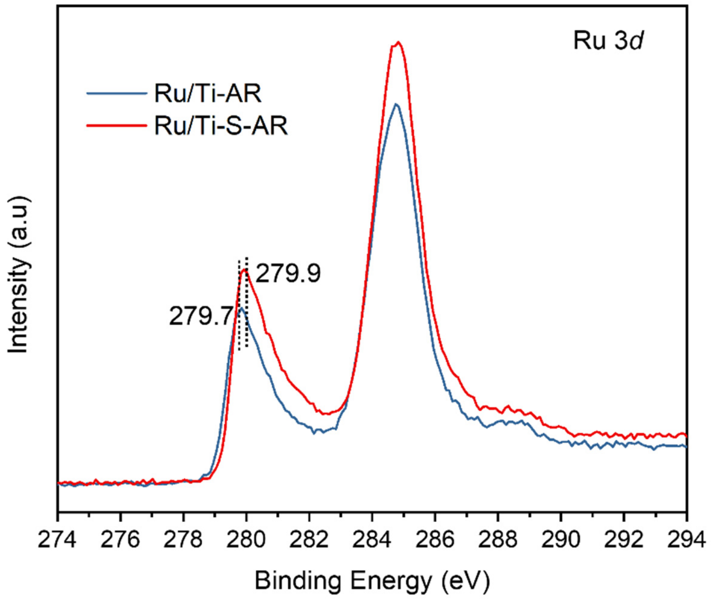

Supplementary Fig. 14 | Ru 3*d* XPS of Ru/Ti-AR and Ru/Ti-S-AR.

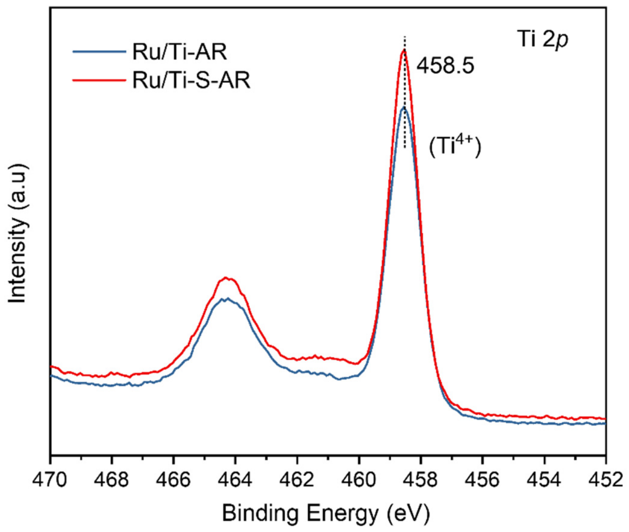

Supplementary Fig. 15 | Ti 2*p* XPS of Ru/Ti-AR and Ru/Ti-S-AR.

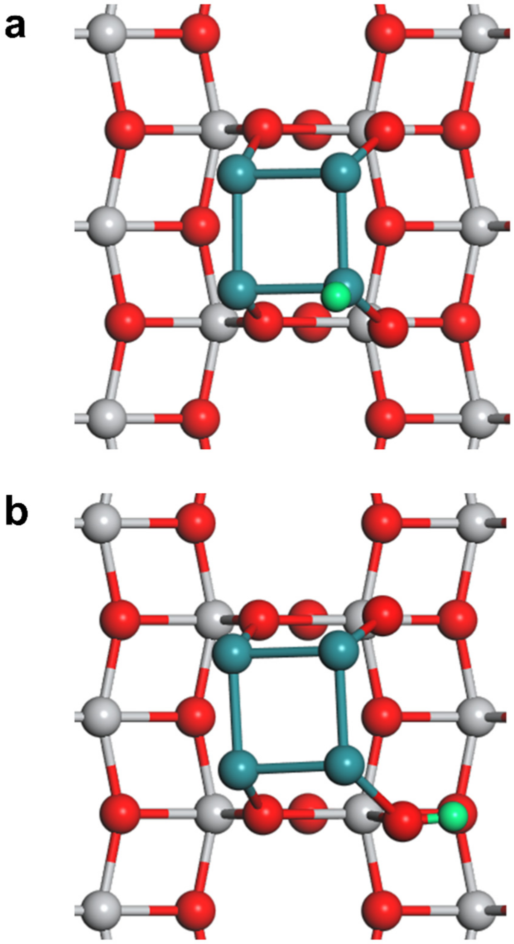

Supplementary Fig. 16 | The surface configuration (**a**) before and (**b**) after H transfer on Ru/Ti-AR (red, O; grey, Ti; cyan, Ru; green, H).

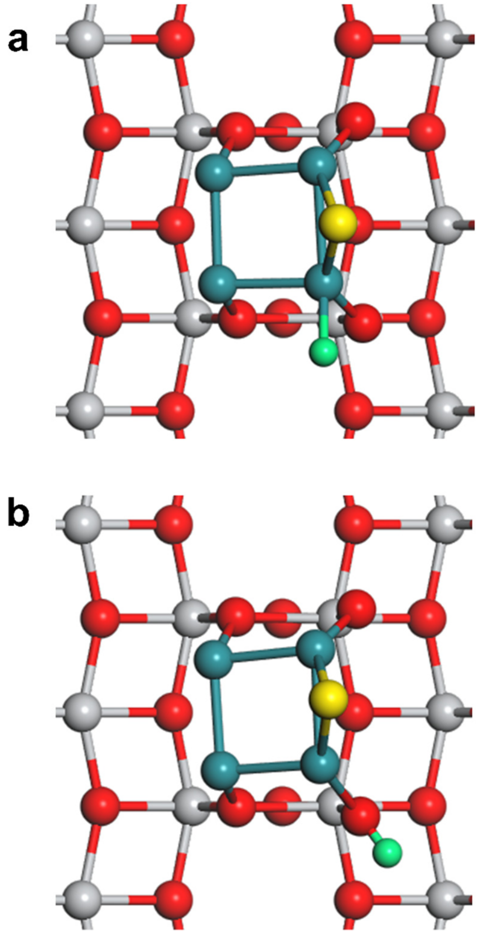

Supplementary Fig. 17 | The surface configuration (**a**) before and (**b**) after H transfer on Ru/Ti-S-AR (red, O; grey, Ti; cyan, Ru; yellow, S; green, H).

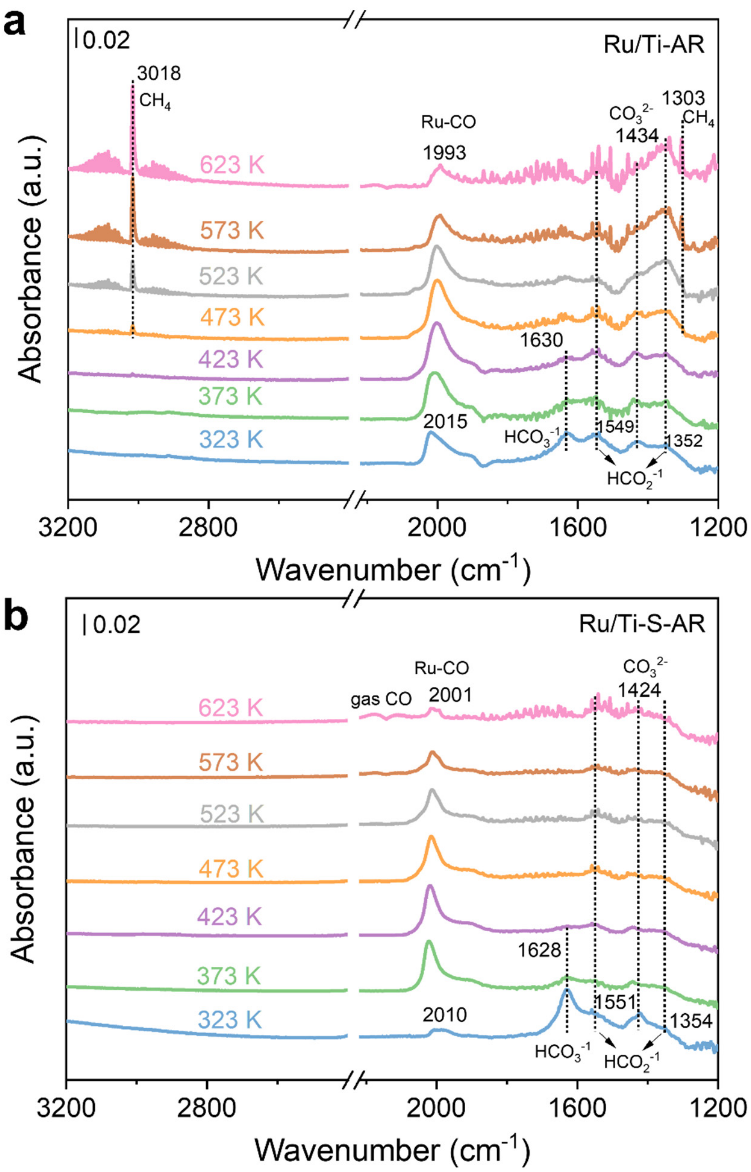

Supplementary Fig. 18 | *In situ* DRIFT spectra following exposure to CO_2 and H_2 at 323 K and subsequent stepwise heating to 623 K for (a) Ru/Ti-AR and (b) Ru/Ti-S-AR.

Note: *In situ* DRIFT spectra under steady-state CO_2 hydrogenation conditions are shown in **Supplementary Fig. 18.** On both Ru/Ti-AR and Ru/Ti-S-AR, CO stretching vibration bands on Ru (2010-2015 cm^{-1}) were observed immediately upon exposure to the feed gas, associated with the appearance of bicarbonates (HCO_3^-, 1630 cm^{-1}), formates (HCO_2^-, 1551 and 1354 cm^{-1}) and carbonates (CO_3^{2-}, 1424 cm^{-1}) species. The bicarbonates were less stable and disappeared completely at 473 K. The intensity of the bands of formates and carbonates exhibited a limited change above 373 K. Additionally, it was observed that the intermediate CO adsorbed at Ru site of Ru/Ti-AR could be converted to CH_4 (3018 cm^{-1}) when the reaction temperature was

above 473 K, while the intermediate CO adsorbed at Ru site of Ru/Ti-S-AR was stable and no CH_4 was obtained.

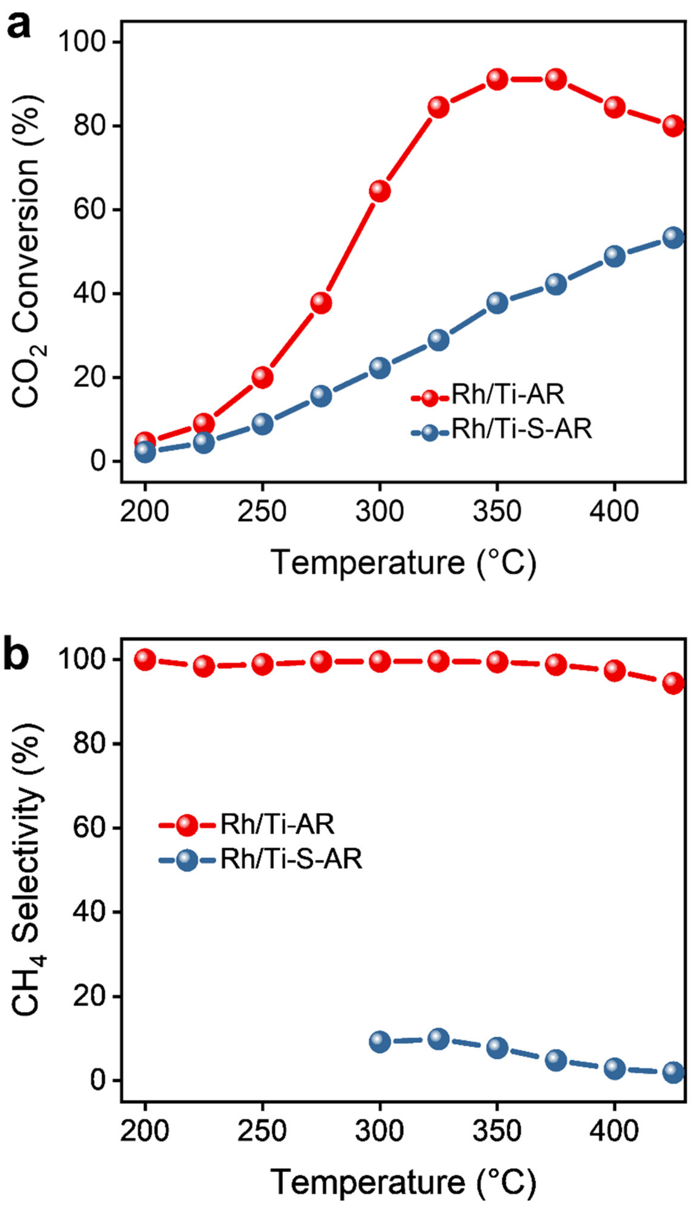

Supplementary Fig. 19 | Temperature-dependent **(a)** CO_2 conversions and **(b)** CH_4 selectivity on Rh/TiO_2 catalysts containing or not containing sulfate species.

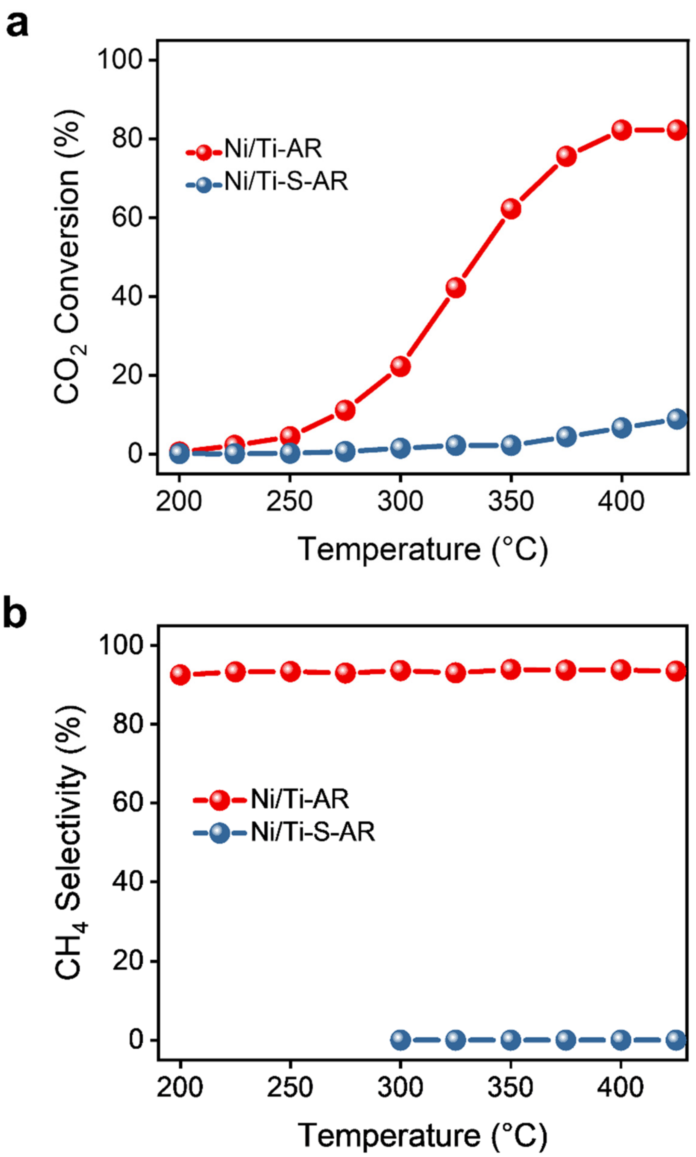

Supplementary Fig. 20 | Temperature-dependent **(a)** CO_2 conversions and **(b)** CH_4 selectivity on Ni/TiO_2 catalysts containing or not containing sulfate species.
